# Estimating the responses of winter wheat yields to moisture variations in the past 35 years in Jiangsu Province of China

**DOI:** 10.1371/journal.pone.0191217

**Published:** 2018-01-12

**Authors:** Xiangying Xu, Ping Gao, Xinkai Zhu, Wenshan Guo, Jinfeng Ding, Chunyan Li

**Affiliations:** 1 Jiangsu Provincial Key Lab of Crop Genetics and Physiology/Wheat Research Institute, Yangzhou University, Yangzhou, Jiangsu Province, China; 2 Meteorological Bureau of Jiangsu Province, Nanjing, Jiangsu Province, China; Institute of Genetics and Developmental Biology Chinese Academy of Sciences, CHINA

## Abstract

Jiangsu is an important agricultural province in China. Winter wheat, as the second major grain crop in the province, is greatly affected by moisture variations. The objective of this study was to investigate whether there were significant trends in changes in the moisture conditions during wheat growing seasons over the past decades and how the wheat yields responded to different moisture levels by means of a popular drought index, the Standardized Precipitation Evapotranspiration Index (SPEI). The study started with a trend analysis and quantification of the moisture conditions with the Mann-Kendall test and Sen’s Slope method, respectively. Then, correlation analysis was carried out to determine the relationship between de-trended wheat yields and multi-scalar SPEI. Finally, a multivariate panel regression model was established to reveal the quantitative yield responses to moisture variations. The results showed that the moisture conditions in Jiangsu were generally at a normal level, but this century appeared slightly drier in because of the relatively high temperatures. There was a significant correlation between short time scale SPEI values and wheat yields. Among the three critical stages of wheat development, the SPEI values in the late growth stage (April-June) had a closer linkage to the yields than in the seedling stage (October-November) and the over-wintering stage (December-February). Moreover, the yield responses displayed an asymmetric characteristic, namely, moisture excess led to higher yield losses compared to moisture deficit in this region. The maximum yield increment could be obtained under the moisture level of slight drought according to the 3-month SPEI at the late growth stage, while extreme wetting resulted in the most severe yield losses. The moisture conditions in the first 15 years of the 21^st^ century were more favorable than in the last 20 years of the 20^th^ century for wheat production in Jiangsu.

## 1. Introduction

Global warming may change the frequency and quantity of precipitation, which would consequently affect the water availability for crop production of the world [1]. Winter wheat, as one of the two major staple food crops in China, is greatly affected by water supply [2–4]. In the North China Plain (NCP), one of Chinese most important agricultural regions, it becomes more difficult to maintain and achieve further productivity increases due to severe drought risk [5]. Jiangsu Province has an intersection with the southern part of the NCP and becomes more important in wheat production in China because the northern part of the NCP suffered wheat abandonment in recent years and there is a north-to-south transition trend for the sown area of wheat across the NCP [6].

In Jiangsu, winter wheat is the second major cereal crop accounting for approximately 30% of the total grain output. According to the Jiangsu Statistical Yearbook 2016, the sown area of winter wheat covers 28.13% of the total sown area for farm crops after rice, which is 29.59%. With the growing population and scaling living standards due to economic development, the importance of wheat production, especially the stability of wheat yields, may increase further [7].

Winter wheat in Jiangsu is grown under rain-supported conditions, is sensitive to moisture variability and has a growing season of approximately nine months (October to June), when crops may suffer from both water deficit and excess. For example, in 2013, the provincial averaged precipitation of April is much less than the normal level. In the northern areas, precipitation is 20%~70% of the normal level and in the southern area, it is 40%~90% of the normal level [8]. In contrast, in 2015, the provincial average precipitation of April was 38% more than the normal level and was even 90% beyond the normal in individual regions [9]. Meanwhile, a number of studies have noted that the moisture conditions of Jiangsu have markedly changed during the past decades [10–13]. The changing frequency, duration and intensity of precipitation combined with the warming temperatures may have a great influence on wheat yields, which gives urgency to the studies of yield responses in this area.

Although many efforts have been made to evaluate the actual effects of moisture variations on wheat yield formation, the influence of inter-annual moisture variations on wheat yields and its evolution remain elusive, especially at regional or national scales [3, 14]. The traditional long-term evaluation of wheat yield responses to moisture is based on the precipitation variations, either through a process-based crop model or an observation-based statistical model, because precipitation is the main variable determining the drought and wet conditions and the data are attainable in most regions. However, the agricultural wetness or drought is a condition of sufficient or insufficient moisture caused by precipitation over some time period, respectively [15]. The span of time from the arrival of precipitation until water is available as soil moisture or groundwater may take months, seasons, or even years. The lagged and cumulative effects of precipitation on crops should not be neglected [16]. For this reason, multi-scalar drought indices have been developed to quantitatively evaluate the moisture conditions in agriculture, meteorology and hydrology during the last decades [17–19].

Among these indices, the landmark is the Palmer drought severity index (PDSI), which was proposed by W. C. Palmer in 1965 based on years of meteorological data from the Midwest of the United States [20]. It enables the measurement of both dryness and wetness based on a soil water balance equation that incorporates multiple parameters, such as precipitation, runoff, available water content of soil, evaporation, soil water recharge, water loss from the soil, etc. The main shortcomings of the PDSI are related to its fixed temporal scale, difficulty in interpreting, complex calculation, as well as multiple input variables that are not readily available [21, 22]. Although the variations of the PDSI have improved the original index and are used extensively in different regions of the world, the inherent complexity limited their smart application [22, 23].

A relatively simple index, the Standardized Precipitation Index (SPI), was proposed by McKee et al. in 1993. It provides the measurement of drought and wet events with negative (drought) and positive (wet) values similar to PDSI [15]. The difference is that it only requires one input variable, precipitation, which simplifies the interpretation, and is able to quantify precipitation deficits or surplus on multiple time scales which are determined by different applications [24–26]. Although it was designed for drought detecting and monitoring, it has also been used to monitor wetter-than-normal conditions in several studies. For example, Seiler et al. (2002) used the SPI to monitor flood risk in the southern Cordoba Province in Argentina and obtained satisfactory results [24]. Du et al. (2013) verified the usefulness of the SPI in drought/flood monitoring in Hunan Province, China [27]. However, the main criticism of the index is that it only considers precipitation data, neglecting the importance of other variables, especially temperature, in the present warming circumstances [16].

A two-parameter index called the Standardized Precipitation Evapotranspiration Index (SPEI) was developed by Vicente-Serrano et al. (2010) to give a better representation than SPI with an improved capability to identify drought [16, 28]. The SPEI incorporates the potential evapotranspiration (PET) as a driving factor of water availability and the calculation is similar to that of SPI, and it has been claimed to have combined the advantages of the two previous indices: the PDSI and the SPI [15, 20, 28, 29]. The SPEI has been widely used in recent years, and the performance has been proven to be satisfactory in diverse studies [30–37].

Several recent studies have been conducted using the SPEI to evaluate the long-term yield responses of crops. For example, Vicente-Serrano et al. (2012) investigated global wheat yields with six different drought indices and found stronger yield–drought correlations for the SPEI than for the other indices. Ming et al. (2015) conducted research on the drought impact on maize yields in NCP based on the SPEI with an improved calculation method of PET by the Penman-Monteith equation. They concluded that the 3-month SPEI in August is highly correlated with the de-trended yield in the study area. Potopová et al. (2011, 2015, 2016) assessed the drought-induced decline in crop harvest in the Republic of Moldova and the Czech Republic by using the SPEI. They found differences in the responses of crops to various lags of the SPEI. On these bases, our objectives are to determine the SPEI-yield relationship and estimate the wheat yield responses to moisture by a statistical model.

With the availability of high-quality data, statistical methods play an important role in evaluating the impacts of climate change on crop yields [38]. Although a statistical model is not so accurate as a process-based model in describing input parameters, such as cultivated varieties and soil properties, it is able to explain the yield responses qualitatively and quantitatively with accurate observed data to provide support for yield prediction and disaster prevention. Numerous studies have used statistical models for crop yield assessment in specific regions and acquired valuable results [1, 39, 40]. For example, Wu et al. (2004) developed a drought risk-assessment model for corn and soybeans in Nebraska, USA and obtained a reliable assessment with more than 80% possibility [41]. Lobell et al. (2014) applied a cross-sectional regression model between maize yield and daytime vapor pressure deficit (VPD) with USDA data and the results agree with the simulated yield responses of the Agricultural Production Systems Simulator (APSIM) model [39].

In this study, we established a statistical regression model with provincial panel data to unravel the quantitative relationship between wheat yields and moisture conditions of the key wheat growing stages by the SPEI in Jiangsu. The purpose of our study is to figure out the evolution of moisture conditions and its influence on wheat yields over the past 35 years. The results may provide references to field management and policy development with the overall aim of ensuring the stable production of winter wheat in Jiangsu.

## 2. Materials and methods

### 2.1 Study area

Jiangsu Province is located on the eastern coast of China, between 116°18’E-121°57’E longitude and 30°45’N-35°20’N latitude, belonging to the flat Yangtze River Delta. The province covers an area of 107,200 km^2^ and has a high density population of approximately 70 million, making food supply a major concern in this area. The climate of the province has features of the monsoon. The southern area belongs to a subtropical humid monsoon climate, while the northern area belongs to a warm temperate humid monsoon climate. The average annual temperature is 13°C to 16°C and annual rainfall is 724 mm to 1210 mm with an obvious regional difference.

### 2.2 Data

Some of the meteorological data were collected from the National Meteorological Information Center of the China Meteorological Administration [42]. Meteorological stations are shown in Table 1. The winter wheat yield data (1980–2014) were collected from the agricultural economic statistics database of the Ministry of Agriculture of the People’s Republic of China and the local statistical yearbooks.

**Table 1:** Location of meteorological stations.

Station	Latitude & Longitude	Station	Latitude & Longitude
**Ganyu**	34°30’N,119°04’E	**Yangzhou**	32°15’N,119°15’E
**Xuzhou**	34°10’N,117°05’E	**Nantong**	32°03’N,120°35’E
**Xuyi**	32°35’N,118°19’E	**Nanjing**	32°00’N,118°29’E
**Dongtai**	32°31’N,120°11’E	**Liyang**	31°16’N,119°17’E
**Gaoyou**	32°29’N,119°16’E	**Wujiang**	31°05’N,120°22’E

The phenological period of winter wheat at each site was determined by references from experts and other researchers’ reports [10, 43], as well as the main crop growth period database of the National Agricultural Scientific Data Sharing Center [44]. Because the calculation of SPEI is based on monthly precipitation and monthly mean temperature as input parameters, the key growing stages of winter wheat in Jiangsu are coarsely determined as the sowing and seedling stage (S1, Oct.-Nov.), over-wintering stage (S2, Dec.-Feb.) and late growth stage (S3, Apr. -Jun.).

### 2.3 Trend test

In this study, the temporal evolutions of the considered precipitations and SPEI variables were determined using the Mann-Kendall method, which is a widely used nonparametric rank-based test recommended by the World Meteorological Organization. Since it is efficient against non-normal underlying distributions and robust in eliminating the influence of extremes, this method is superior to parameter tests, particularly in the case of meteorological time series [45]. Suppose X (x_1_, x_2_, x_3_, … x_n_) is a time series. The null hypothesis H0 of no trend is rejected if the statistic is significantly different (p < 0.05) from zero [46]. When the alternative hypothesis H1 is assumed, time series X has a significant change trend. The calculation is performed using Eqs (1) ~ (5). At first, the S value of the sequence X is computed:

$$S\;=\;\sum_{k\;=\;1}^{n-1}\sum_{j\;=\;k+1}^{n}sgn(x_{j}-x_{k})$$ (1)

where x_K_ and x_j_ are values at time k and j in the time series (j > k), respectively. The symbol function sgn is defined as follows:

$$\text{sgn}\left(x_{j}-x_{k}\right)\;=\{\begin{array}{c} +1,\;x_{j}-x_{k}>0\\ 0,\;\;x_{j}-x_{k}\;=\;0\\ -1,\;x_{j}-x_{k}<0 \end{array}$$ (2)

When the sample size n is larger than 10, the S statistic is considered to be the approximately normal distribution. The expectation and variance of S are:

E(S) = 0 (3)

$$\text{Var}\left(\text{S}\right)=\frac{1}{18}\left[n\left(n-1\right)\left(2n+5\right)-\sum_{i}e_{i}\left(e_{i}-1\right)\left(2e_{i}+5\right)\right]$$ (4)

where e_i_ is the extent of any given tie, which referred to the number of variables that are repeated. Then, calculate the Z value of the standard normal distribution:

$$\text{Z}=\{\begin{array}{ll} \frac{S-1}{\sqrt{Var\left(S\right)}}, & S>0\\ 0, & S=0\\ \frac{S+1}{\sqrt{Var\left(S\right)}},\; & S<0 \end{array}$$ (5)

When$\;|Z|\geq Z_{\frac{\alpha }{2}}$, we reject H0 and accept H1 at a significant level of α. In this study, α is equal to 0.05. If the value of Z is positive, the sequence has an upward trend; otherwise, if Z is negative, it has a downward trend. Nevertheless, the Mann-Kendall test does not offer information about the magnitude of the trend. Hence, the Sen’s slope estimator is adopted to calculate the slope of the trend by Eq (6).

$$Slope\;=\;median(\frac{x_{j}-x_{k}}{j-k})$$ (6)

Similarly, x_K_ and x_j_ are values at time k and j (j > k), respectively, and Slope is the median of the time series calculated.

### 2.4 SPEI calculation

Vicente-Serrano et al. (2010a) presented the detailed calculation process of the multi-scalar SPEI in their article. The executable file of the calculation that we used is freely available online [47]; meanwhile an R package maintained by Beguería et al. is also available [48]. The calculation is mainly divided into three steps [16]:

1. Calculate the monthly difference *D*_*i*_ between the precipitation *P*_*i*_ and potential evapotranspiration *PET*_*i*_ for the month *i*:
  $$D_{i}\;=\;P_{i}-{PET}_{i}$$ (7)
  Here, the *PET*_*i*_ is calculated by the Thornthwaite (1948) method based on the monthly average temperature and latitude of the site following the method of Vicente-Serrano et al. (2010a). Then, the cumulative *D* sequences are obtained by using *D*_*i*_ values at different time scales.
2. The Log-Logistic probability distribution F(x) is used to fit the aggregated *D* sequence, in which the probability density function *f(x)* is calculated as follows:
  $$f(x)\;=\;\frac{\beta }{\alpha }{\left(\frac{x-\gamma }{\alpha }\right)}^{\beta -1}{\left[1+{\left(\frac{x-\gamma }{\alpha }\right)}^{\beta }\right]}^{-2}$$ (8)
  where *α*, *β* and *γ* are the scale, shape and origin parameters, respectively. These parameters can be obtained following the L-moment procedure, and then the probability distribution function F(x) is calculated.
3. With F(x), the SPEI can easily be obtained as the standardized values of F(x). The SPEI is given by:
  $$SPEI\;=\;W-\frac{C_{0}+C_{1}W+C_{2}W^{2}}{1+d_{1}W+d_{2}W^{2}+d_{3}W^{3}}$$ (9)
  where
  $$W\;=\;\sqrt{-2\text{ln}\;\left(P\right)},\;P\leq 0.5$$ (10)
  Here, P = 1-F(x). If *P* > 0.5, then *P* is replaced by 1−*P*. The constants are *C*_0_ = 2.515517, *C*_1_ = 0.802853, *C*_2_ = 0.010328, *d*_1_ = 1.432788, *d*_2_ = 0.189269 and *d*_3_ = 0.001308.

As an index of water balance, SPEI can be used to indicate the degree of drought and wetness. The drought and wet levels based on SPEI values are displayed in Table 2 [49].

**Table 2:** Drought and wet levels based on SPEI values.

Level	SPEI value
**Extreme drought**	≤ −2.0
**Moderate drought**	−1.99~−1.0
**Slight drought**	−0.99~−0.5
**Normal**	−0.49~0.49
**Slight wetting**	0.5~0.99
**Moderate wetting**	1.0~1.99
**Extreme wetting**	≥ 2.0

### 2.5 Statistical analyses

To detect the SPEI-yield linkage, a correlation analysis was carried out between wheat yields and the mean SPEI values of growing seasons at multiple time scales using R software. As a time series, the trend was removed from the wheat yield to eliminate bias due to non-climate factors [50, 51]. We used the first order difference to get stationary time series of yields and SPEI [1]. The Pearson’s correlation coefficient was applied to measure the strength of the association between de-trended yield and de-trended SPEI with a significance threshold of p < 0.05.

We constructed a multivariate regression model based on the provincial level panel data to quantify the responses of yields to moisture variations. With two dimensions, the panel data provides more information than time series or cross-sectional data. It enables control for time-invariant unobserved heterogeneity and improves the estimation efficiency in statistical analysis [52]. Therefore, panel data models are often used in modeling crop yields [38, 40]. With balanced panel data of 350 (10 sites and 35 years) observations, we established a panel regression model:

$$Y_{i,t}\;=\;\beta_{i}+\alpha_{1}{SPEI}_{i,t}+\alpha_{2}{SPEI}_{i,t}^{2}+\alpha_{3}t+u_{i,t}$$ (11)

where Y_*i*,*t*_ and SPEI_*i*,*t*_ are the yields and SPEI values at site *i* in year *t*. Several candidate SPEI values at different time scales are compared, which will be detailed in section 3.2, and we will select the most relevant SPEI values as the explanatory variables in this model. The quadratic term is included to acquire the nonlinear responses of yields to drought or wet conditions. The explanatory variable *t* is a linear time trend that removes the effects of technological improvements on wheat yields over time [52]. β_i_ is the intercept of site *i* denoting the time-invariant features of each site, such as the soil type, cultivation custom and management characteristics. u_i,t_ is the random error term. Because it includes the unobservable factors, we conducted a DW (Durbin-Watson) test to detect the autocorrelation of residuals. When autocorrelation exists, the autoregressive term AR(1) will be added to the panel model. *α*_1_, *α*_2_ and *α*_3_ are time-invariant slope coefficients indicating the constant influences of explanatory variables to the explained variable. The parameter estimation of the model is implemented by using Eviews with the least squares dummy variable (LSDV) method. Here, we estimate an individual fixed effects model, since site fixed effects β_i_ may be correlated with SPEI_i,t_, violating the assumption of a random effects model that fixed effects need to be orthogonal to the other covariates of the model [52].

## 3. Results

### 3.1 Trend analysis

To understand the relationship between winter wheat yields and the moisture conditions during growing seasons (Oct. to Jun.), it is necessary to analyze the trends between yields and SPEI. First, the precipitation trends and SPEI trends were analyzed to detect the evolution of water input and water balance over years using the Mann–Kendall and Sen’s slope tests. Then, the de-trended yields were computed to eliminate the increasing trends of wheat yields caused by technique improvements and obtain the annual yield fluctuations caused by climate, known as the climate-determined yield.

#### 3.1.1 Precipitation trend

**Fig 1:**
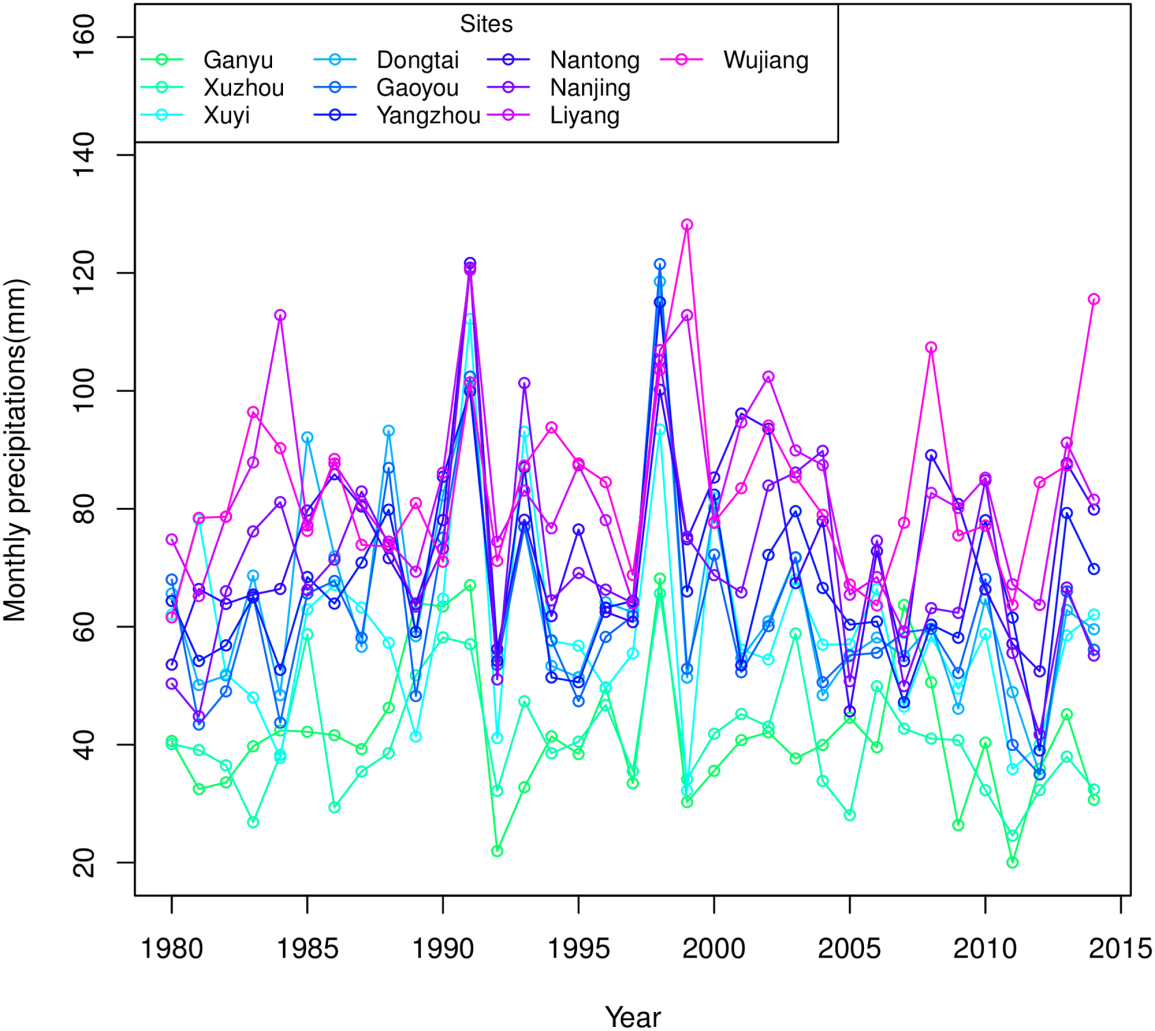
Monthly precipitations of 10 sites in Jiangsu Province during the growing seasons (Oct.-Jun.) from 1979 to 2014.

The monthly total precipitations of wheat growing seasons were calculated from 1979 to 2014. Fig 1 illustrates that precipitations in the northern sites (Ganyu and Xuzhou) are less than those in the southern sites, indicating the uneven distribution of precipitation in Jiangsu. Moreover, the precipitations exhibit periodic fluctuations every 5 to 7 years, especially in the southern sites. The Sen’s slopes reveal that downward trends existed in most of the series, except Nantong and Wujiang. However, all the trends of 10 sites are not significant by applying the Mann-Kendall trend detection method. We compared the monthly precipitations for the periods of 1980–1989, 1990–1999 and 2000–2014, respectively, and found that in 1990s, the average monthly precipitation was approximately 14% more than 1980s and 2000s, while in the other two periods, it exhibited almost the same amount. The results indicate that although empirically and practically, the frequency and intensity of the precipitations may have some changes in recent years, the average monthly amount of the rainfall still fluctuates within a normal range during the wheat growing seasons in Jiangsu.

#### 3.1.2 SPEI trend

**Fig 2:**
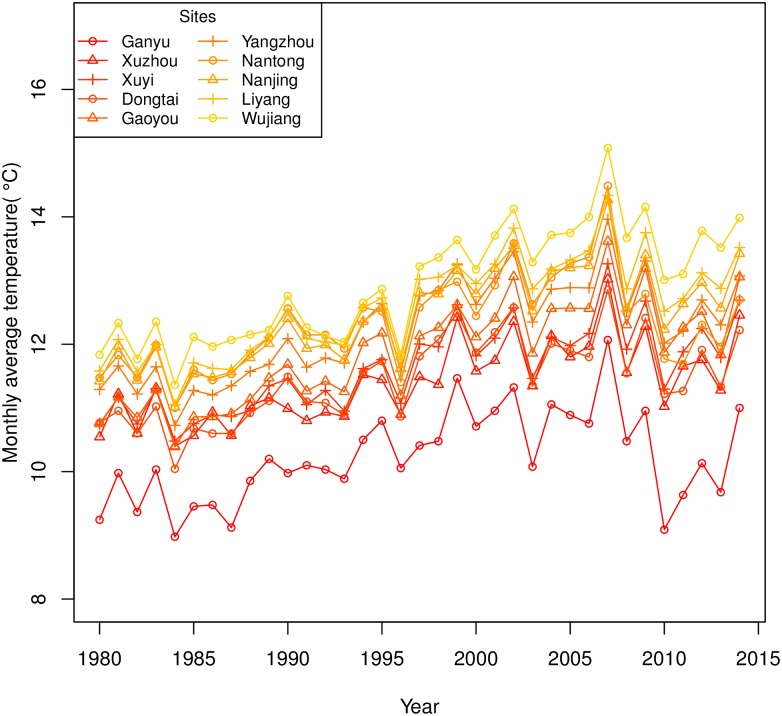
Monthly average temperatures of 10 sites in Jiangsu Province during the growing seasons (Oct.-Jun.) from 1979 to 2014.

The monthly average temperatures were calculated during the October-June period for 10 sites from 1979 to 2014. According to the result of the Mann-Kendall test, the temperatures of 10 stations showed consistent significant upward trends (p < 0.001). Using Sen’s slope to quantify the trends, we found that the slopes ranged from 0.04°C/year to 0.07°C/year, and the minimal value occurred in Ganyu, the northern site, while the maximal value occurred in Wujiang, the southern site. The variations of the mean temperatures are illustrated in Fig 2. The apparent temporal trends in temperature during the wheat growing seasons increase the potential evapotranspiration, and then the water demand for crops [16]. Hence, droughts that lead to low crop yields may become more extensive and intense [53]. Therefore, the SPEI is more appropriate than other drought indices for this study as a measure to quantify the long-term moisture conditions.

**Fig 3:**
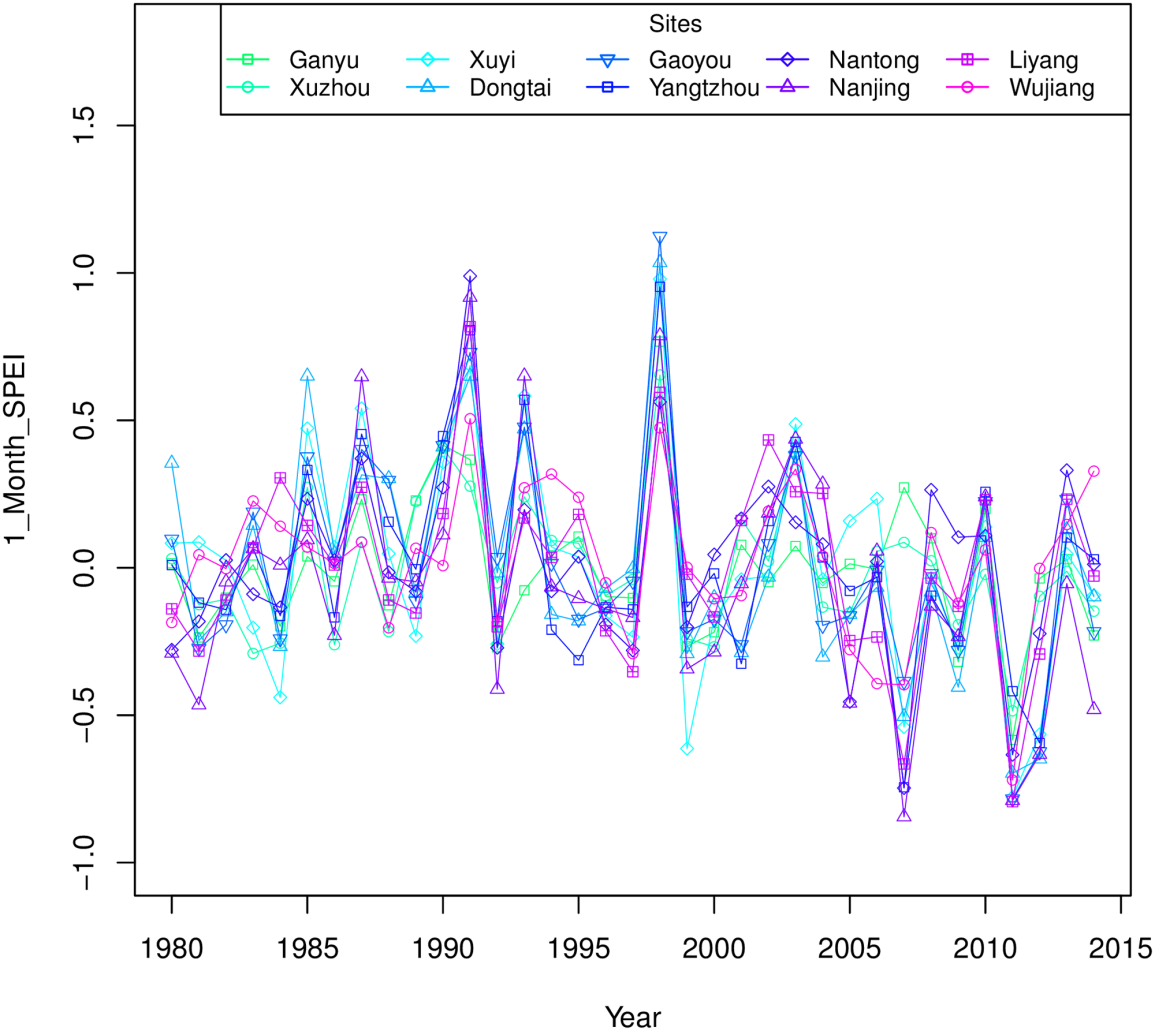
Average 1_month SPEI of 10 sites in Jiangsu Province during the growing seasons (Oct.-Jun.) from 1979 to 2014.

**Fig 4:**
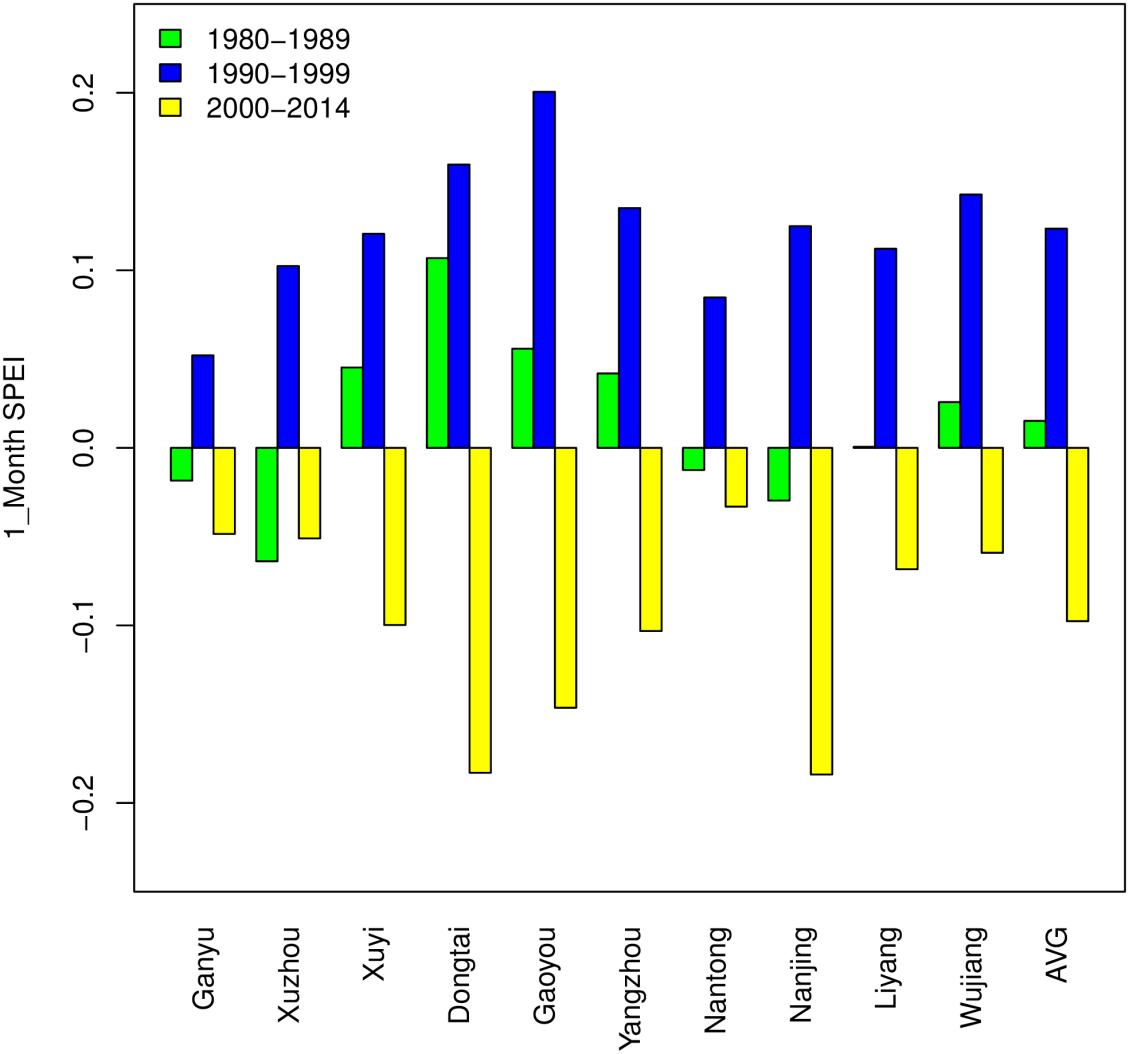
Average 1_month SPEI of 10 sites in Jiangsu Province during the growing seasons (Oct.-Jun.) from 1980–1989, 1990–1999 and 2000–2014. Note: AVG means the average 1_month SPEI values of 10 sites.

Fig 3 depicts the evolution of the average SPEI on a one-month scale in 10 sites. According to the Mann-Kendall test and Sen’s slope, there are no significant trends among most of the sites except for Dongtai (p < 0.05, slope = −0.014/year), and the slopes of all sequences are negative except for Nanjing (slope = 0.001/year). However, the SPEI fluctuations of most sites show a low level after the year 2000. To investigate the relative levels of different decades, average moisture levels of the periods 1980–1989, 1990–1999 and 2000–2014 are calculated in terms of 1_month SPEI in Fig 4. It shows that the average moisture conditions are under normal levels in all three periods, though in the years after 2000, the SPEI values of all sites are below zero, indicating the lower levels in moisture than before. Note that there are obvious differences in SPEI values between 1980s and 2000s. The reason is mainly related to the high temperatures in this century. Although several recent modeling studies have found that a “hiatus” of the warming appeared in the recent years of this century [54], the average temperature from 2000–2014 is 1.32°C higher than that in the 1980s according to the records of 10 sites. Hence the corresponding PET is larger in the 2000s; meanwhile, the precipitation does not increase significantly, which makes it reasonable that Jiangsu got a little drier in this century than in the previous years.

#### 3.1.3 Winter wheat yield trend

**Fig 5:**
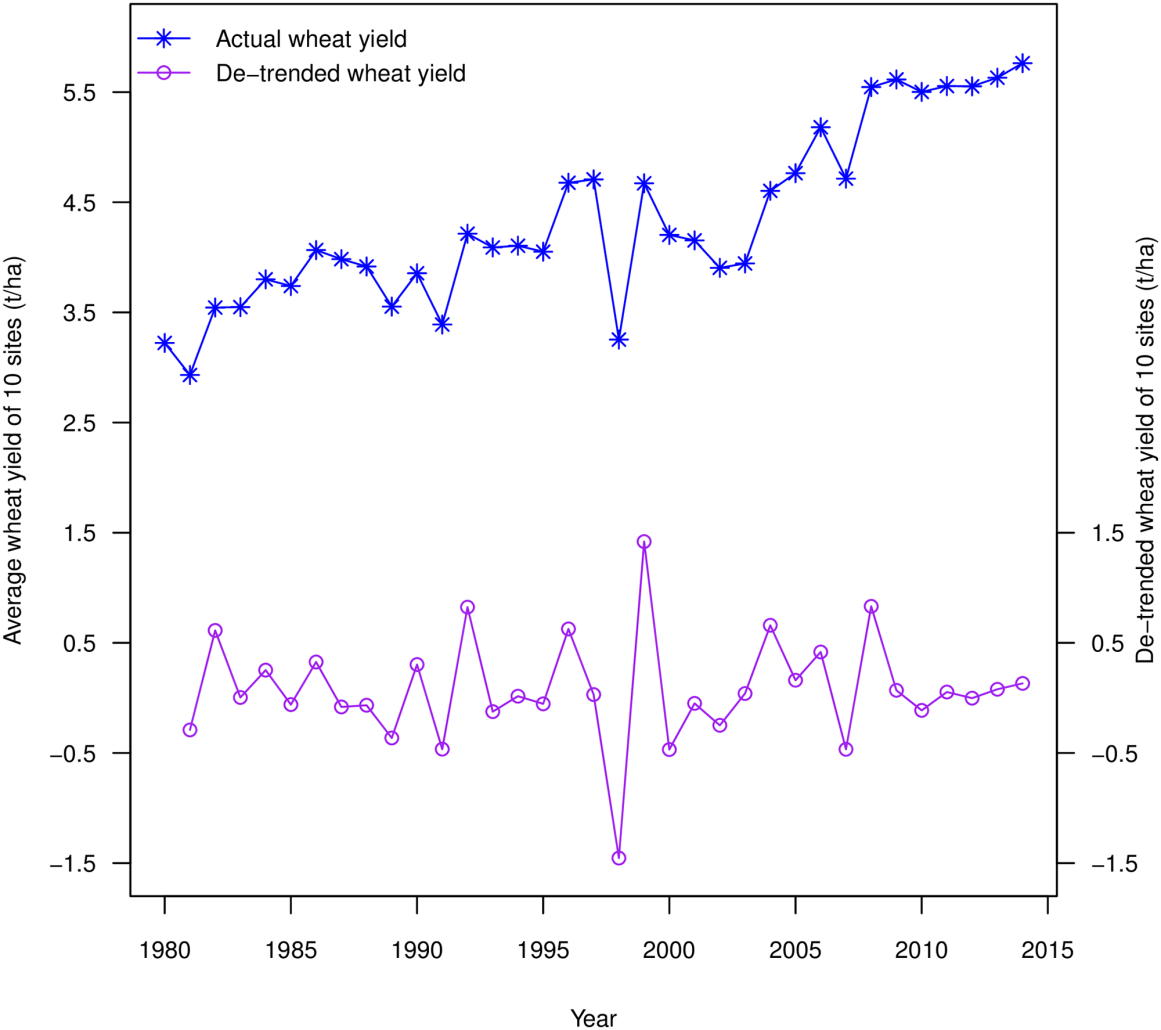
The 10-site average winter wheat yields from 1980 to 2014 and the 10-site average de-trended yields from 1981 to 2014 in Jiangsu Province.

The yield of winter wheat in Jiangsu has obviously increased in the last 35 years. The average yields of 10 sites ranged from 2.93 t/ha in 1981 to 5.76 t/ha in 2014. However, the wheat yield is influenced by many non-climate factors, such as the new varieties, the frequency of fertilizer application, the improved field management, etc. To eliminate bias from these factors and obtain the climate-determined yield variability, the trend was removed using first order differences. The yields from 1980 to 2014 and the de-trended yields from 1981 to 2014 are provided in Fig 5.

### 3.2 SPEI-wheat yield linkage

Agricultural drought (wet) is related to soil moisture and typically has a short time scale [15]. Thus, we selected four different time scales (1, 3, 6 and 9 months) to calculate the SPEI values. The use of growing season averages for climate variables is common in statistical approaches [38]. Hence, the mean SPEI values from October to June were analyzed. The results of correlation analysis are shown in Table 3, where the Pearson correlation coefficients between de-trended yields and de-trended SPEI values are computed.

**Table 3:** The Pearson correlation coefficients between yield and growing season averaged SPEI.

Station	SPEI_1	SPEI_3	SPEI_6	SPEI_9
**Ganyu**	-0.36*	-0.23	0.09	0.31
**Xuzhou**	-0.48**	-0.47**	-0.21	0.22
**Xuyi**	-0.35*	-0.34*	-0.18	0.001
**Dongtai**	-0.27	-0.20	0.06	0.33*
**Gaoyou**	-0.42**	-0.39*	-0.25	-0.02
**Yangzhou**	-0.37*	-0.35*	-0.20	-0.01
**Nantong**	-0.33*	-0.32	-0.21	-0.01
**Nanjing**	-0.35*	-0.29	-0.006	0.25
**Liyang**	-0.48**	-0.44**	-0.19	0.11
**Wujiang**	-0.42**	-0.38*	-0.31	-0.09

SPEI_1, SPEI_3, SPEI_6 and SPEI_9 represent 1-, 3-, 6- and 9-month SPEI values, respectively. * * and * indicate statistical significance at 1% and 5% confidence levels, respectively.

The 1-month SPEI represents the average moisture condition of the growing season and the 3-month SPEI reflects the seasonal moisture characteristics [50]. Results show that 1 and 3-month SPEI closely correspond to most of the yields in the 10 sites. While the 6-month and 9-month SPEI indicate moisture conditions of the half year and the whole growing season, both representing medium-term moisture variability. Table 3 indicates that they had no pronounced correlation with wheat yields in most of the sites. It implies that short-term SPEI values are greatly correlated with the rain-fed wheat yields because they reflect the soil water content, which influences the water balance of the crop, physiological-biochemical mechanism and then the nutrition, growth and yield of crops. Meanwhile, the negative correlation coefficients indicate that the main limitation factor of wheat yields in Jiangsu is the wet stress.

The 1 and 3-month SPEI values were selected for further analysis in three key growth stages: sowing and seedling stage (S1), over-wintering stage (S2) and late growth stage (S3). As shown in Table 4, there is a significant correlation between yields and 3-month SPEI at S3 in most of the sites, indicating that moisture at this stage had a greater effect on wheat yields than in the earlier stages. The result is consistent with a large number of agricultural practices, which have proven that the late growth stage, especially the heading and grain formation period, is the key yield determinant period, and under favorable conditions, approximately 70% to 90% of the carbohydrates in grains are formed during this period [55, 56]. Either too much or too little water will reduce the root activity, resulting in a decrease of the grain number per spike and grain weight. Therefore, SPEI values of S3 are appropriate for the regression analysis of yields.

**Table 4:** The Pearson correlation coefficient between de-trended yield and de-trended of 1 and 3-month SPEI during three key growth stages.

Station	SPEI_1_S1	SPEI_1_S2	SPEI_1_S3	SPEI_3_S1	SPEI_3_S2	SPEI_3_S3
**Ganyu**	0.14	-0.33	-0.24	0.14	-0.27	-0.16
**Xuzhou**	0.08	-0.37*	-0.30	0.07	-0.37*	-0.39*
**Xuyi**	0.23	-0.23	-0.25	-0.13	-0.23	-0.41*
**Dongtai**	0.27	-0.18	-0.41*	-0.07	-0.06	-0.49**
**Gaoyou**	0.24	-0.23	-0.42*	0.20	-0.33	-0.42*
**Yangzhou**	0.14	-0.24	-0.26	0.09	-0.26	-0.40*
**Nantong**	-0.12	-0.37*	-0.25	-0.13	-0.25	-0.42*
**Nanjing**	0.21	-0.24	-0.28	0.1	-0.20	-0.38*
**Liyang**	0.21	-0.50**	-0.14	0.15	-0.39*	-0.34*
**Wujiang**	-0.04	-0.58**	0.07	-0.20	-0.45**	-0.1

SPEI_1 and SPEI_3 represent 1 and 3-month SPEI values, respectively. S1, S2 and S3 represent the sowing and seedling stage (S1), over-wintering stage (S2) and late growth stage (S3), respectively. * * and * indicate statistical significance at 1% and 5% confidence levels, respectively.

As shown in Table 4, in Ganyu and Wujiang, the SPEI_3_S3 values are not significantly related to yields, indicating that the yields of the two sites are little affected by the changes in moisture. This is due to the fact that the two sites have particular climatic conditions. Ganyu is located on the north-east coast of Jiangsu, where the moisture conditions are favorable, and it is part of the so-called “northern granary” in Jiangsu. A similar result was mentioned by Wu et al. (2012) in their article, who concluded that the waterlogging stress of this area is rare in spring. Conversely, Wujiang, located in southern watery region, has a humid climate in spring and low wheat yields. The annual variations in moisture changed the level of wetness but did not change the situation of water excess that resulted in the sustained low level of yields, so moisture fluctuations in spring had less effects on wheat yields in this area. Moreover, both 1 and 3-month SPEI values of S2 had very significant negative effects on yields in the southern sites, Liyang (r = -0.5 and -0.39) and Wujiang (r = -0.58 and -0.45), because the rainy weather in winter would aggravate the waterlogging and influence the growth of the seedlings, and substantially affect the yields. Therefore, at the southern sites, wet stress is more influential in the over-wintering stage than in the late growing stage.

### 3.3 Regression model for yield responses

The average 3-month SPEI values of the late growth stage (SPEI_3_S3) were chosen to establish the fixed effects panel regression model for their high relevance to wheat yields. By the DW (Durbin-Watson) test, we detected that autocorrelation existed and the AR(1) term is added to the panel model representing some neglected autocorrelation factors, such as the policy influences. Thus, the non-climate factors are effectively controlled, and the yield responses can be directly presented by different moisture levels. The coefficients of the model are shown in Table 5.

**Table 5:** Estimation results for the panel model.

Variable	Coefficient	Fixed effects	Coefficient	Fixed effects	Coefficient
**${\bar{\beta }}_{i}$**	3.3376	**β_Ganyu**	0.2166	**β_Yangzhou**	0.2799
**SPEI_3_S3**	-0.1592	**β_Xuzhou**	0.3275	**β_Nantong**	-0.0369
**SPEI_3_S3**^**2**^	-0.1297	**β_Xuyi**	-0.1691	**β_Nanjing**	-0.6264
**t**	0.0626	**β_Dongtai**	0.5424	**β_Liyang**	-0.7786
**AR(1)**	0.3900	**β_Gaoyou**	0.7057	**β_Wujiang**	-0.4612

All explanatory variables have reached a significance level of p = 0.0000 and the model resulted in an adjusted *R*^2^ of 0.75. It suggests that 75% of the yield changes are explained, which exceeds the fitting degrees of the models proposed by Ming et al. (2015) for maize yields in NCP. The average intercept of the sites ${\bar{\beta }}_{\text{i}}$ is 3.3376 and the negative coefficients of SPEI_3_S3 and its quadratic term imply a parabola relationship between yield and SPEI_3_S3. The fixed effects of each site fluctuate from -0.7786 to 0.7057 around ${\bar{\beta }}_{\text{i}}$, reflecting the specific adjustment in yield for each site. The panel model implies an optimum inferred SPEI value of -0.61 and the threshold values denoting yield losses are 0 and -1.23, neglecting the influences of other factors. The yield responses to different moisture levels at the late growth stage are depicted in Table 6. It indicates that wetting conditions of all three levels tend to incur wheat yield losses, and a negative relationship between yield and SPEI_3_S3 appears. When the moisture level falls into extreme wetting, the yield losses may reach more than 837 kg/ha on average, which indicates the severe yield decline at Xuyi and Nantong in 1991 and at Dongtai and Gaoyou in 1998. Conversely, in the case of slight drought, it tends to produce slightly higher yields, though the increment is very limited from 29.5 kg/ha to 47.2 kg/ha, while, both moderate and extreme drought exhibit a positive yield-SPEI relationship, reflecting that water deficit causes yield losses. Under normal moisture levels, the wheat yields in Jiangsu fluctuate weakly from -112.03 kg/ha to 47.18 kg/ha. The results show an asymmetry in yield responses, namely, at the same levels of drought and wet (slight, moderate or extreme), wet conditions lead to more remarkable yield losses than drought according to SPEI_3_S3.

**Table 6:** Wheat yield responses to SPEI_3_S3.

level	SPEI_3_S3	Yield response (kg/ha)
**Extreme wetting**	≥2.0	≤-837.2
**Moderate wetting**	[1.0, 2.0)	(-837.2, -288.9]
**Slight wetting**	[0.5, 1.0)	(-288.9, -112.0]
**Normal**	(-0.5, 0.5)	(-112.0, 47.2)
**Slight drought**	(-1.0, -0.5]	(29.5, 47.2]
**Moderate drought**	(-2.0, -1.0]	(-200.4, 29.5]
**Extreme drought**	≤-2.0	≤-200.4

**Fig 6:**
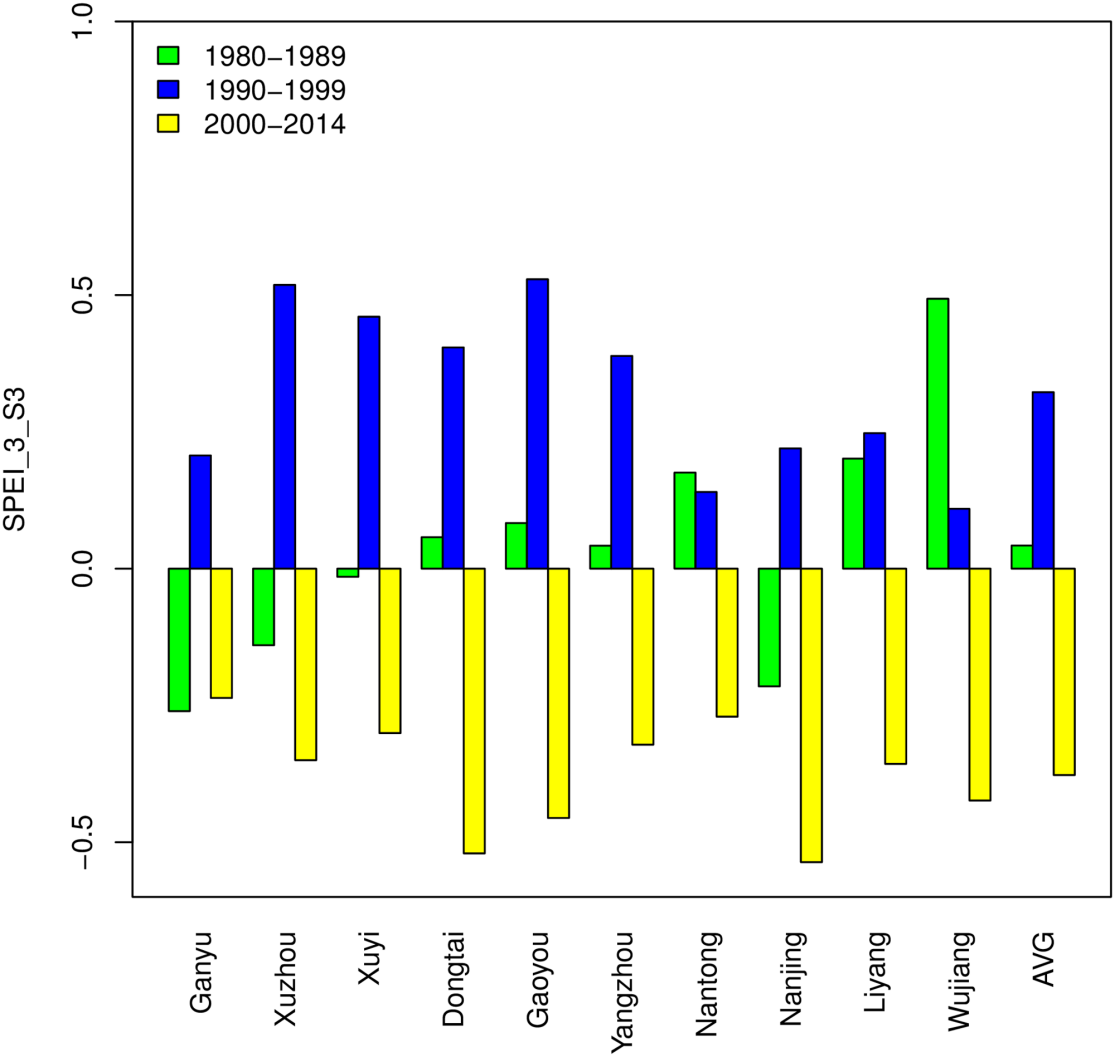
Average 3-month SPEI of 10 sites in Jiangsu Province during late growth stage (April-June) from 1980–1989, 1990–1999 and 2000–2014. Note: AVG means the average SPEI_3_S3 values of 10 sites.

To investigate the yield responses of different decades, SPEI_3_S3 values are divided into three groups: 1980–1989, 1990–1999 and 2000–2014. As shown in Fig 6, the average SPEI_3_S3 values of all sites are positive in the 1990s and negative in this century, and the mean values of each decade are 0.3225 and -0.3772. In the 1980s, it is in the middle, with a mean value of 0.0422. The corresponding average yield responses (kg/ha) in the 1980s, 1990s and 2000s are -6.95, -64.83 and 41.60, respectively. This suggests that the drying tendency that appeared in the critical growing stage (April-June) in this century contributes to the promotion of wheat yields. According to the optimum inferred SPEI value of the panel model, there is still a gap between the present moisture conditions and the most favorable situations.

## 4. Discussion

In recent decades, the increasing trend of temperature in Jiangsu has been so evident that it is likely to interact with precipitation to affect agricultural production in ways that change water availability [57]. The trend analysis has shown that the changing trend in moisture is not as significant as that in temperature within Jiangsu. It supports the concerns related to Lobell et al. (2011), in that in the most regions of the world, the warming effects are much more important than the effects of rainfall trends. By means of the popular drought index, SPEI, as a measure to quantify the moisture conditions, we detected lower moisture levels during wheat growing seasons in this century than in the years before 2000, which reveals that future impacts in the moisture variations of Jiangsu will be mainly driven by high temperatures rather than variations in precipitations. Nonetheless, we only consider the average growing season conditions and ignore the extreme rainfall and temperature events, which may have short-term impacts on moisture variations that influence wheat yields.

The correlation analysis showed there is a close relationship between de-trended yield and de-trended SPEI values at 1 and 3-month scales. It verifies that rain-fed crop yields are mainly related to short time-scale SPEI values, which directly reflect the agricultural moisture conditions for the reason that the climatic water balance calculated to obtain SPEI is related to water absorption by roots, as well as transpiration by the leaves of the crops. The short time scales are consistent with the key phenological phases, during which crops are sensitive to moisture conditions. A similar result has been provided by Potopová et al. (2015), who found that winter wheat yield is significantly affected by a severe drought in May-June at a 1-7-month lags of SPEI in the Czech Republic. Additionally, despite the differences in crop strains and research areas, they found that SPEI has strong associations with other crops at critical growth stages. As a result, the methods used in this study are applicable for other rain-fed crops, such as maize, barley, soybean, and sorghum, to study the yield responses in different moisture conditions.

The detailed analysis in three key growth stages exhibited a pronounced correlation between yield and SPEI_3_S3. This suggests that the yield responses vary in different growth stages and the late growth stage is the most sensitive period to moisture variability. This agrees with the results obtained by Wu et al. (2016) in this region, who suggested that the risk of waterlogging in the middle and late stages of wheat growth is higher than that in the earlier stages. Apart from the different physiological characteristics of each growth period, the stage differences in yield responses are partly associated with the remedial measures taken in the following stages. When winter wheat is exposed to a disaster in earlier stages, it may have time to remedy itself and the disaster will not directly incur yield losses. Therefore, during early growth stages, it is non-negligible to take measures, such as fertilization, irrigation and drainage by farmers and agricultural technology personnel when wheat is under drought or waterlogging. More importantly, prophylactic measures, such as improving drainage systems, should be provided to prevent yield reduction before late growth stages, because most of the final grain yields are derived from the photosynthate made in these periods, and there is limited time for remedy when agricultural disaster appears.

The panel model reveals that if other factors are fixed, the maximum yield increment could be obtained under slight drought, which reflects the moisture excess of the wheat growing seasons due to the geographic and climatic characteristics of this area. As a low-lying coastal province, Jiangsu has a shallow groundwater depth and high humidity, especially in the rainy spring when winter wheat is at the late growth stage. The appearance of water excess increases the risks of waterlogging and diseases, such as rust and scab, which bring further reductions in the yields [58]. On the other hand, the slight drought during this period helps to decrease the groundwater level to a suitable depth, which increases the yield potential. However, when the groundwater depth declines below the threshold by more severe drought stress, the wheat yields will suffer a reduction due to water shortage. The asymmetric yield responses imply a weak adaptability of wheat production to water surplus in this region over past years. Nevertheless, the low level of moisture in the first 15 years of this century is favorable and helpful to the improvement of wheat yields.

Our research provides an application of the drought index SPEI in a humid and semi-humid region to monitor the level of agricultural moisture and to explain the fluctuations of wheat yields. With increasingly accurate weather forecasts and the development of climate-smart agriculture, the SPEI will be used more extensively and efficiently in the field of agricultural moisture assessment. The methods and outcomes of this study can be used by researchers, government sectors and organizations to evaluate the recent changes in agro-meteorological situations and their tendency with respect to the expected climate change. Additionally, it provides references to agricultural disaster prevention and policy development to guarantee the sustainable increase of agricultural production in China.

## Supporting information

S1 File The SPEI values of ten sites in Jiangsu Province. (XLSX)
